# Risk factors and a predictive model for the occurrence of adverse outcomes in patients with new-onset refractory status epilepsy

**DOI:** 10.3389/fnmol.2024.1360949

**Published:** 2024-04-16

**Authors:** Qiuyan Luo, Rong Lai, Miao Su, Zichao Wu, Huiyu Feng, Hongyan Zhou

**Affiliations:** ^1^Neurological Intensive Unit, Department of Neurology, The First Affiliated Hospital, Sun Yat-sen University, Guangzhou, China; ^2^Department of Neurology, Guangzhou Woman and Children’s Medical Centre, Guangzhou, China

**Keywords:** new-onset refractory status epilepsy, neurocritical illness, predictive factor, nomogram, autoimmune mechanisms

## Abstract

**Objectives:**

To determine risk factors for the occurrence of adverse outcomes in patients with new-onset refractory status epilepsy (NORSE) and to construct a concomitant nomogram.

**Methods:**

Seventy-six adult patients with NORSE who were admitted to the Department of Neurology, First Affiliated Hospital of Sun Yat-sen University between January 2016 and December 2022 were enrolled for the study. Participants were divided into two—those with good and poor functional outcomes—and their pertinent data was obtained from the hospital medical recording system. Univariate analysis was used to identify potential causes of poor outcomes in both groups and a multivariate logistic regression model was used to identify risk factors for the occurrence of poor outcomes. Using the R programming language RMS package, a nomogram was created to predict the occurrence of poor outcomes.

**Results:**

The NORSE risk of adverse outcome nomogram model included four predictors, namely duration of mechanical ventilation (OR = 4.370, 95% CI 1.221–15.640, *p* = 0.023), antiviral therapy (OR = 0.045, 95% CI 0.005–0.399, *p* = 0.005), number of anesthetics (OR = 13.428, 95% CI 2.16–83.48, *p* = 0.005) and neutrophil count/lymphocyte count ratio (NLR) (OR = 5.248, 95% CI 1.509–18.252, *p* = 0.009). The nomogram had good consistency and discrimination in predicting risk and can thus assist clinical care providers to assess outcomes for NORSE patients. Through ordinary bootstrap analyses, the results of the original set prediction were confirmed as consistent with those of the test set.

**Conclusion:**

The nomogram model of risk of adverse outcomes in NORSE adult patients developed in this study can facilitate clinicians to predict the risk of adverse outcomes in NORSE patients and make timely and reasonable interventions for patients at high risk of adverse outcomes.

## 1 Introduction

Epilepsy is a chronic brain disorder that, although with many underlying causes, is generally characterized by recurring, episodic and temporary abnormalities of the central nervous system due to excessive neuronal discharges. An epileptic condition is status epilepticus (SE) (Trinka et al., 2015) where a seizure either lasts for more than 5 min or the patient does not regain consciousness between seizures—this is the second most frequent neurological emergency (Hesdorffer et al., 1998). Approximately 40% of SE patients are unresponsive to first- and second-line treatments (Holtkamp et al., 2005). An uncommon but challenging form of SE is new-onset refractory status epilepticus (NORSE), which is characterized by not only persistent refractory epilepticus in people with no prior history of epilepsy but also unresponsiveness to standard treatment (Hirsch et al., 2018). Its etiology remains unknown.

While some treatable causes, such as autoimmune encephalitis, have been identified in a subset of patients with New-Onset Refractory Status Epilepticus (NORSE), leading to significant research interest, the majority of patients remain cryptogenic (52%) (Sculier and Gaspard, 2019). Furthermore, there is limited knowledge regarding the potential pathophysiological mechanisms underlying NORSE, and the outcomes of treatments are often poor. Research indicates that adverse outcomes in NORSE patients are associated with several factors, including the duration of seizures, mechanical ventilation, and the utilization of antiepileptic drugs, with the prognosis frequently being extremely poor (Towne et al., 1994; Holtkamp et al., 2005; Hocker et al., 2013; Sutter et al., 2013; Chen et al., 2021). Given that NORSE is a rare clinical presentation, there are no literature statistics available on its incidence rate; however, the incidence rate of Refractory Status Epilepticus (RSE) is reported to be between 3.4 and 7.2 per 100,000 individuals annually, with NORSE accounting for 20% of these cases (Mantoan Ritter and Nashef, 2021). A meta-analysis of 193 RSE patients from 1980 to 2001 reported a mortality rate of 48% (Claassen et al., 2002). It is therefore critical to rapidly and objectively assess the condition and mortality risk in NORSE patients.

Currently, there are three scoring systems for Status Epilepticus internationally: the Status Epilepticus Severity Score (STESS), the Epidemiology-based Mortality Score in Status Epilepticus (EMSE), and the END-IT score. However, a meta-analysis published by Yuan et al. on the predictive scoring of Status Epilepticus suggests that the effectiveness of the STESS, EMSE, and END-IT scores in clinical use remains questionable, highlighting the need for further research to develop more accurate scoring systems (Yuan et al., 2023). This underscores that predicting the functional outcomes of patients with Status Epilepticus remains a challenge, especially for the rarer type such as New-Onset Refractory Status Epilepticus (NORSE), for which no studies have yet proposed a related outcome prediction system.

Given the uncertain prognosis of NORSE, treatment decisions are contentious and challenging, particularly regarding the duration of maintenance therapy during prolonged seizures, which remains a subject of debate. Against this backdrop, we conducted this study to identify prognostic predictors for NORSE, with the aim that these findings will assist clinicians in formulating treatment strategies to improve the prognosis of NORSE, facilitating the enhancement of care quality and the efficient utilization of medical resources.

Nomograms integrate and visualize influencing factors so as to predict clinical events on an individual basis. Thus, to aid clinicians to identify NORSE patients who are at a high risk of experiencing negative outcomes, this study aimed to analyze the factors influencing the occurrence of adverse outcomes in NORSE patients, by creating a nomogram model.

## 2 Methods

### 2.1 Study participants

Seventy-six NORSE adult patients who were admitted to the Department of Neurology, First Hospital of Sun Yat-sen University from January 2016 to December 2022 were enrolled for the study. All met the diagnostic criteria for NORSE (Gaspard et al., 2018) as defined at the 1st International Workshop on New-Onset Refractory Persistent Epilepsy (NORSE) and Febrile Infection-Related Epilepsy Syndrome (FIRES) Symposium. These were: an exhibition of multiple clinical syndromes; no previous history of epilepsy nor related neurological conditions; first-time presentation; attainment of refractory status epilepticus; and no identified structural, toxic nor metabolic etiology. Patient functional outcome at discharge was assessed by the modified Rankin Scale (mRS) score (Balu et al., 2019)—good functional outcome was defined as an mRS score ≤ 2, ranging from no disability (mRS = 0) to a slight disability but able to independently care for themselves (mRS = 2) whereas poor functional outcome (defined as mRS >2) represented a range from moderate disability that requires assistance for daily living (mRS = 3) to severe disability that requires ongoing care (mRS = 5) and possibly causes death (mRS = 6). This was used to divide patients into poor (mRS >2, *n* = 52) and good (mRS ≤2, *n* = 24) functional outcome groups.

**Figure 1:**
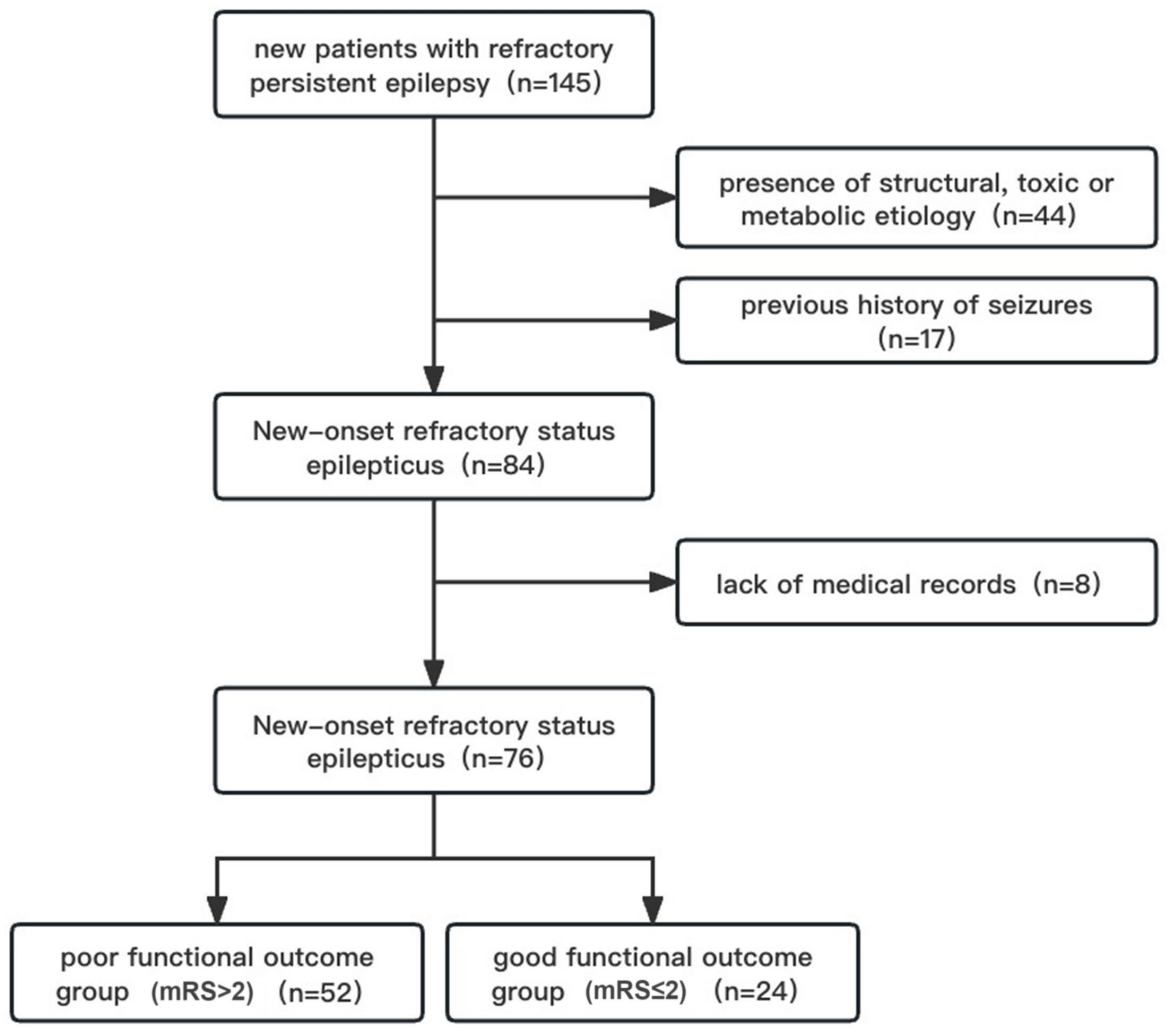
Case screening flow diagram.

NORSE patients who met the following criteria were enrolled in the study: new patients with refractory persistent epilepsy; age of onset ≥18 -year-old; and admission to the Department of Neurology, First Affiliated Hospital of Sun Yat-sen University. Exclusion criteria were: a previous history of seizures; identified structural, toxic, or metabolic etiology; lack of medical records due to failure to either continue treatment at the hospital or automatic abandonment (Figure 1).

The study protocol was reviewed and approved by the Ethics Committee of the First Affiliated Hospital of Sun Yat-sen University. It was exempted from obligatory signed informed consent forms from participants as it posed little risk to them.

### 2.2 Data collection

Patient information was obtained from the medical record database of the First Affiliated Hospital of Sun Yat-sen University. This included gender, age, etiology, prodromal stage, presence of infection, intubation, STESS score, END-IT score, GCS (glasglow coma scale), duration on a mechanical ventilator, immunotherapy regimens (one round of hormone shock = 1 g of methylprednisolone for 3 days; one round of IVIg = 0.4 g/kg/d immunoglobulin for 5 days), antiepilepsy regimens, presence of MR lesions, QAlb (Yu et al., 2021) (albumin of CSF/albumin of serum), NLR (Zeng et al., 2019) (neutrophil-to-lymphocyte ratio), CSF pressure of first time, albumin on admission, ICU stay, total hospital stay and financial cost, etc. Indicators were converted to categorical variables using relevant reference ranges.

### 2.3 Statistical analysis

The data were analyzed using SPSS 26.0. The Shapiro-Wilk test was used to perform normality tests. For quantitative data conforming to a normal distribution, comparisons between groups were conducted using independent samples *t*-tests; for quantitative data with a skewed distribution, comparisons between groups were performed using the Mann–Whitney U test. To ensure consistency throughout the document, quantitative data are presented as (x ± s), while count data are presented as case numbers and percentages [n (%)], with comparisons between groups conducted using the chi-square (χ^2^) test. For univariate analysis, an alpha level of 0.10 was used. Multivariate logistic regression was used to analyze factors influencing the occurrence of adverse outcomes in NORSE patients. For multivariate analysis, an alpha level of 0.05 was used. After identifying predictive risk factors with high values for adverse outcomes in NORSE patients, a nomogram for predicting adverse outcomes in NORSE patients was created using the R software RMS package.

### 2.4 Development and validation of the nomogram

To evaluate the predictive ability of the model, the receiver operator characteristic (ROC) curve and the consistency index (C-index) were used. To assess the accuracy of the predictive model, a calibration curve was plotted. The clinical decision curve analysis (DCA) was used to evaluate the clinical validity of the model. Internal validation was performed by bootstrap analysis.

## 3 Results

### 3.1 Patient characteristics

The average age of the 76 NORSE patients was 35.6 ± 19.9 years (median 28.5 years), with 40 (52.6%) males and 36 (47.4%) females and the average length of hospitalization was 41.4 ± 31.6 days (median 31.5 days). The average ICU stay was 24.3 ± 27.8 days and the average cost of hospitalization was ¥256,385.691. At discharge, 52 patients had a poor functional outcome, with a 68.4% incidence of adverse outcomes and 6 in-hospital deaths (7.9%). NCSE was detected by EEG monitoring in 5 patients (6.6%).

Mechanical ventilation was required in 49 of 76 patients (64.5%) with 39 (75%) in the poor outcome group and 10 (41.7%) in the good outcome group. The average duration of mechanical ventilation was 23.2 ± 26.3 days (median 10.5 days) with an average duration of 31.0 ± 28.8 days for mechanical ventilation in the poor outcome group and 6.0 ± 10.1 days for mechanical ventilation in the good outcome group.

All 76 patients underwent third-line coma induction therapy with 37 (48.7%) requiring 2 or more anesthetic agents. The most commonly used anesthetic drug was midazolam (*n* = 52, 68.4%), followed by propofol (*n* = 23, 30.3%), and phenobarbital (*n* = 21, 27.6%).

Of the 76 patients with NORSE enrolled, 39 (51.3%) had autoimmune etiology and 15 (19.7%) had infectious etiology, of which 13 (86.7%) had a diagnosis of viral encephalitis which was mainly diagnosed by combining clinical symptoms, cerebrospinal fluid indices and imaging manifestations. Antiviral therapy was administered to 39 (51.3%) of the 76 patients. It was used in 17 (70.8%) cases in the good outcome group and 22 (42.3%) cases in the poor outcome group.

### 3.2 Risk factors for adverse outcomes

To identify predictors of adverse outcomes, univariate logistic regression was performed for each variable in the development dataset. Variables with *p* < 0.05 included etiology, infection, GCS score, NLR, intubation, mechanical ventilation, days on a ventilator, antiviral therapy, rounds of intravenous immunoglobulin (IVIg), number of anesthetics, number of antiepileptic drugs (AEDs) (Table 1). Statistically significant indicators were included in a multifactorial logistic regression model. The results showed that antiviral therapy, number of anesthetics, mechanical ventilation, and NLR were independent risk factors for adverse outcomes in NORSE patients (*p* < 0.05) (Table 2).

**Table 1:** Univariate logistic regression of risk factors of NORSE.

Variable	Poor functional outcome (*n* = 24)	Good functional outcome (*n* = 52)	χ^2^	*p*-value
Gender			1.756	0.185
Male	15(62.5%)	24(46.2%)		
Female	9(37.5%)	28(53.8%)		
Age			0.033	0.856
≤30	13(54.2%)	27(51.9%)		
>30	11(45.8%)	25(48.1%)		
Etiology			13.639	0.003^**^
Unknown	3(12.5%)	13(25%)		
Autoimmune	8(33.3%)	31(59.6%)		
Infectious	8(33.3%)	7(13.5%)		
Others	5(20.8%)	1(1.9%)		
Premonitory symptom	16(66.7%)	41(78.8%)	1.299	0.254
Prodromal stage			1.383	0.726
≤ 3 days	12(50%)	22(42.3%)		
≤ 1 week	7(29.2%)	15(28.8%)		
≤ 2 weeks	2(8.3%)	3(5.8%)		
>2 weeks	3(12.5%)	12(23.1%)		
GCS score			4.029	0.045^*^
≤8	13(54.2%)	40(76.9%)		
> 8	11(45.8%)	12(23.1%)		
Infection	15(62.5%)	49(94.2%)	10.163	0.001^***^
Intubation	10(41.7%)	39(75%)	7.966	0.005^**^
The duration of mechanical ventilator			18.887	0.000^***^
≤2 weeks	21(87.5%)	19(36.5%)		
≤1 month	1(4.2%)	14(26.9%)		
>1 month	2(8.3%)	19(36.5%)		
Rounds of hormone shock			2.572	0.109
≤1 round	20(83.3%)	34(65.4%)		
>1 round	4(16.7%)	18(34.6%)		
Rounds of IVIg			9.786	0.002^**^
≤1 round	21(87.5%)	26(50%)		
>1 round	3(12.5%)	26(50%)		
Times of plasmapheresis			0.068	0.794
≤5 times	22(91.7%)	45(86.5%)		
>5 times	2(8.3%)	7(13.5%)		
Second-line treatment	3(12.5%)	12(23.1%)	0.588	0.443
Antiviral treatment	17(70.8%)	22(42.3%)	5.349	0.021^*^
Number of AEDs			1.763	0.414
1 kind	7(29.2%)	9(17.3%)		
2 kinds	7(29.2%)	14(26.9%)		
>2 kinds	10(41.7%)	29(55.8%)		
Number of anesthetics			5.392	0.051^*^
1 kind	16(66.7%)	23(44.2%)		
2 kinds	5(20.8%)	9(17.3%)		
>2 kinds	3(12.5%)	20(38.5%)		
Cerebral lesion on MRI	18(75%)	39(75%)	0	1
Cerebral cortex lesion on MRI	11(45.8%)	21(40.4%)	0.2	0.655
Qalb			2.931	0.231
>7.00	9(37.5%)	15(28.8%)		
≤7.00	12(50%)	21(40.4%)		
NLR			5.222	0.022^*^
>4.82	10(41.7%)	36(69.2%)		
≤4.82	14(58.3%)	16(30.8%)		
CSF pressure of first time			0.876	0.349
>180 mm H_2_O	13(54.2%)	34(65.4%)		
≤180 mm H_2_O	11(45.8%)	18(34.6%)		
Albumin on admission			1.236	0.539
<35 g/L	8(33.3%)	22(42.3%)		
35-50 g/L	13(54.2%)	27(51.9%)		
>50 g/L	3(12.5%)	3(5.8%)		

*p < 0.05. **p < 0.01. ***p < 0.001 indicates that the difference is statistically significant.

**Table 2:** Final multivariate logistic regression of risk factors of NORSE.

Variable	β	SE	Wald χ^2^	*P*-value	OR	95% CI	
Etiology	−0.801	0.493	2.646	0.104	0.449	0.171	1.178
The duration of mechanical ventilator	1.475	0.651	5.139	0.023^*^	4.37	1.221	15.64
Rounds of IVIg	1.476	0.878	2.828	0.093	4.375	0.783	24.435
Antiviral treatment	−3.094	1.11	7.772	0.005^**^	0.045	0.005	0.399
NLR	1.658	0.636	6.795	0.009^**^	5.248	1.509	18.252
Number of anesthetics	2.597	0.932	7.761	0.005^**^	13.428	2.16	83.48

*p < 0.05. **p < 0.01. ***p < 0.001 indicates that the difference is statistically significant.

### 3.3 Nomogram model development and validation

**Figure 2:**
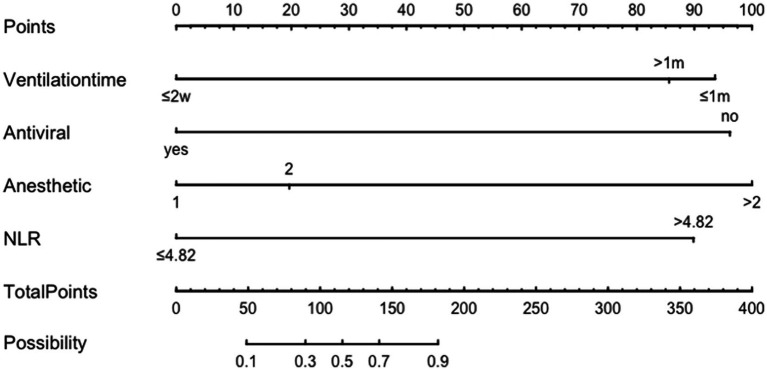
Nomogram model for predicting adverse outcomes in NORSE patients.

The variables identified from the multivariate logistic regression were used as predictor variables and the total score for each factor—the score range was 0–220 and the higher the score, the greater the risk of adverse outcome occurrence—was calculated using the R software RMS package for nomogram analysis (Figure 2). Using the factors of mechanically ventilation, the NLR value, antiviral therapy, the rounds of IVIg, and the number of anesthetics, the corresponding risk of adverse outcomes was calculated by obtaining the corresponding scores of values from the vertical lines on the score line at the top of the column plot, and subsequently adding the scores of all variables to obtain the total score.

The dynamic nomogram: https://luoqiuyan.shinyapps.io/NORSE/.

**Figure 3:**
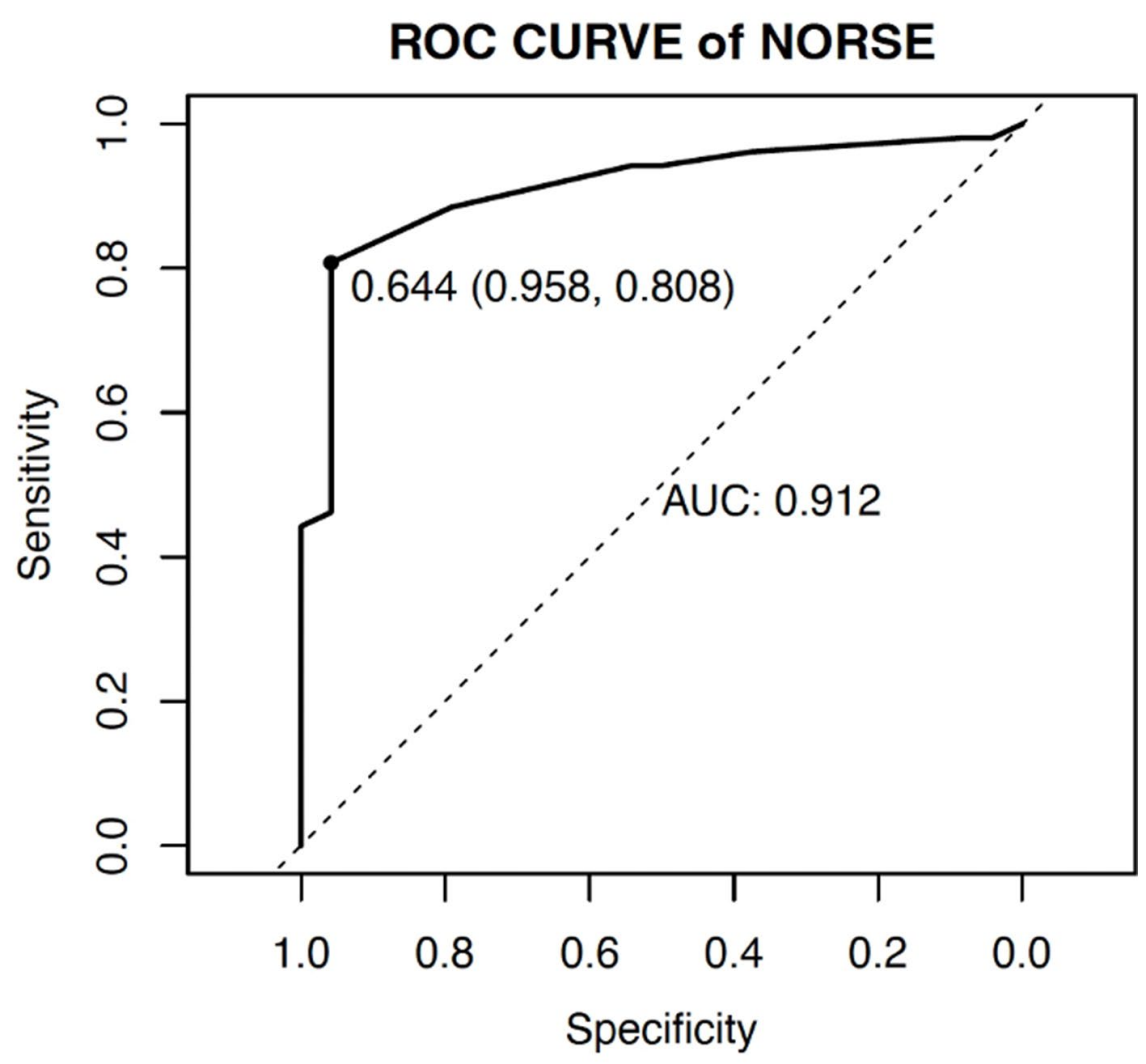
ROC curve of prediction model for adverse outcome in NORSE patients.

The ROC curve was used to determine the ability of the model to predict the occurrence of adverse outcomes in patients with NORSE. The area under the ROC curve (AUC) for this model was 0.912 (95% CI: 0.844–0.980), with a sensitivity of 80.8% and specificity of 95.8% (Figure 3).

**Figure 4:**
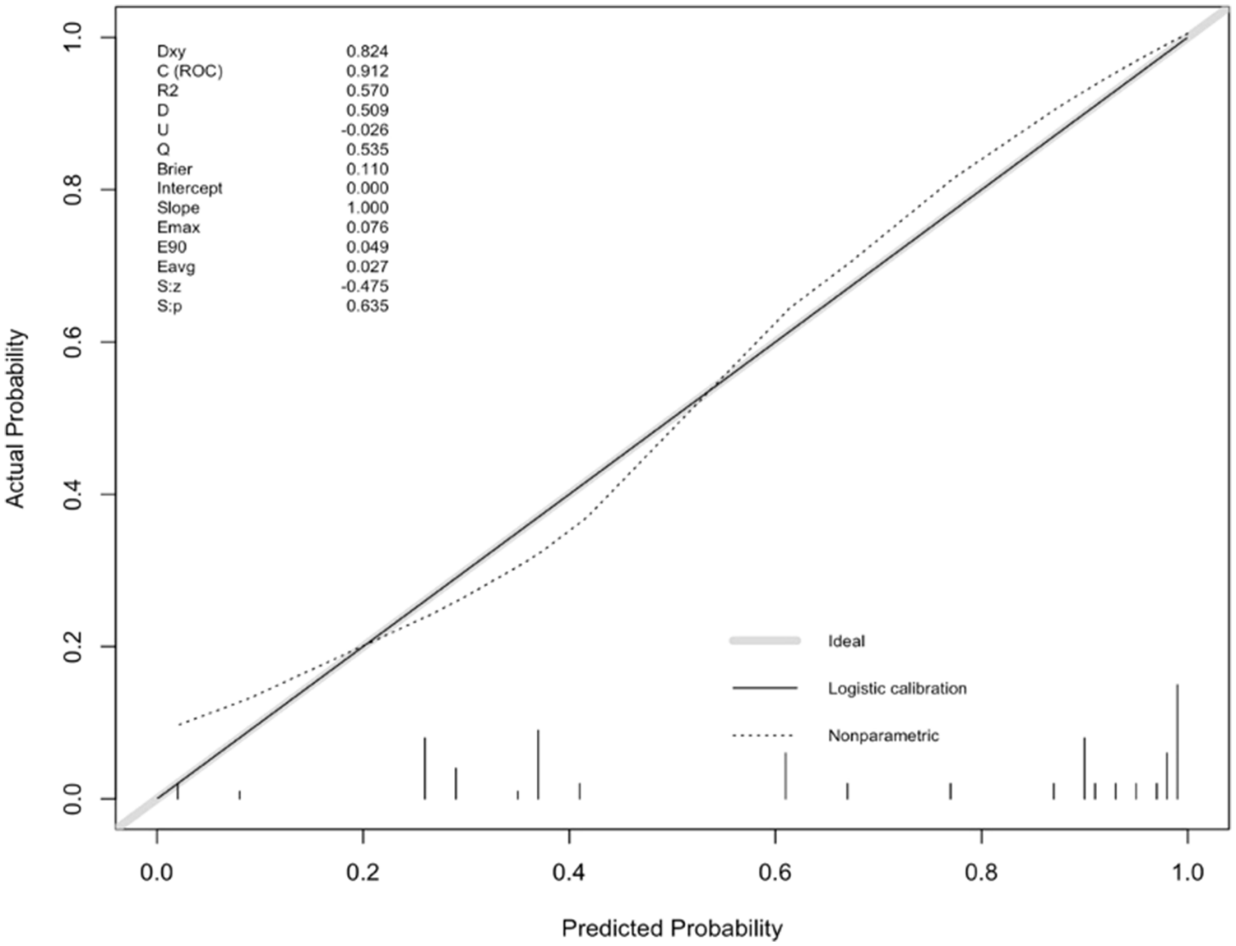
Calibration plot of the nomogram predicting the risk of adverse outcomes versus the actual risk of adverse outcomes.

For internal validation, an analysis of 1,000 bootstrap resamples with replacement was done. Then, the calibration curves for predicted adverse outcomes were plotted against the actual adverse outcomes, with the dashed line indicating the uncalibrated portion and the realization indicating the calibrated portion, both of which were close to the theoretical curve (Figure 4).

**Figure 5:**
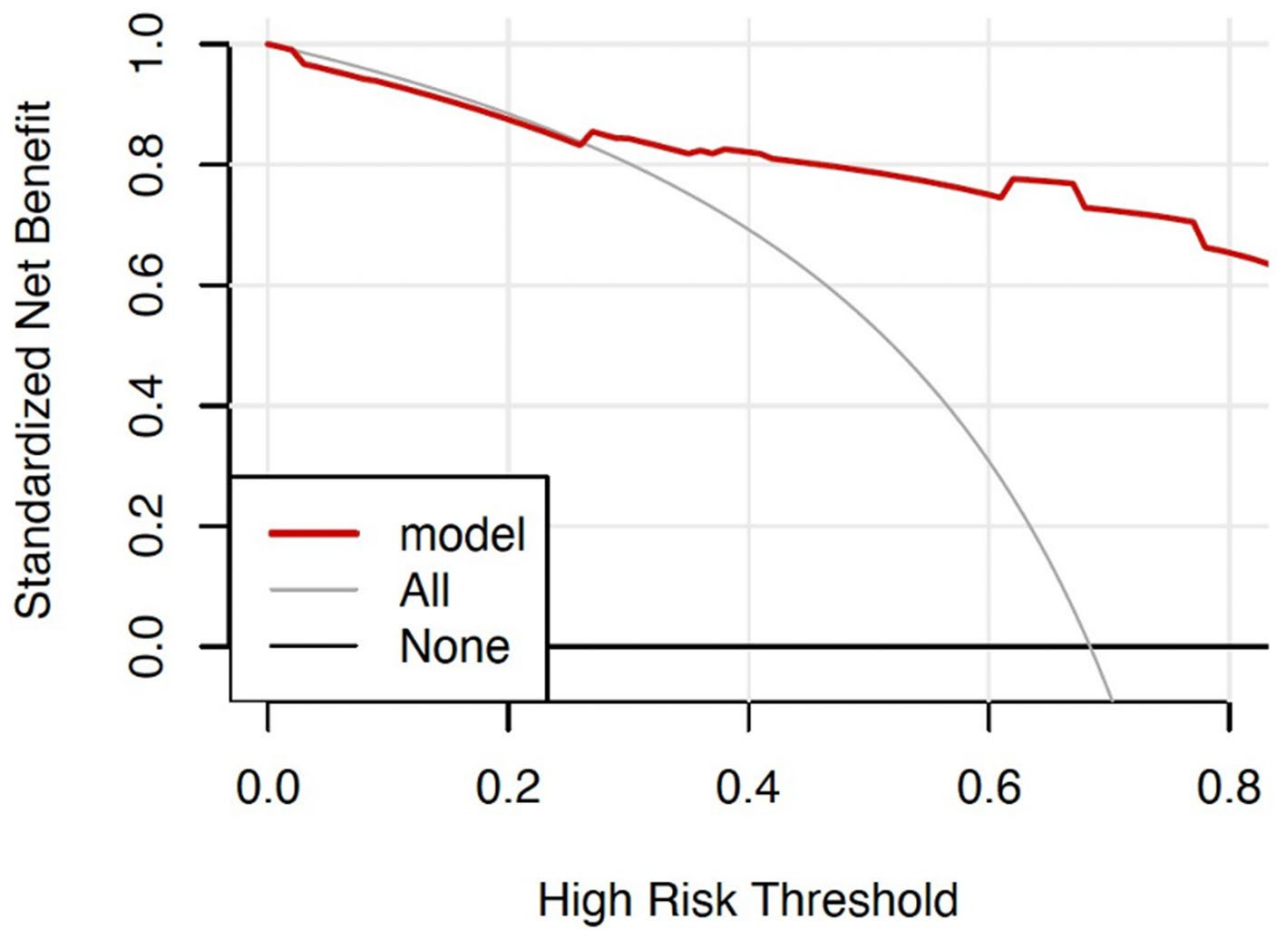
Clinical decision curves for the nomogram predicting adverse outcomes.

For the graph on clinical validity, the thick solid line represented no clinical benefit for patients who do not experience an adverse outcome whereas the thin solid line—an inverse slope with a negative gradient—represented the net clinical benefit for patients who experienced an adverse outcome. The red line above the two extreme curves represented clinical validity. The threshold probability was 25% when the net benefit of patients was higher than the two extreme curves (Figure 5).

Final internal validation was performed via ordinary bootstrapping. The Root Mean Square Error (RMSE) value of the model was calculated to be 0.41.The model fit was moderately high (Alba et al., 2017).

## 4 Discussion

New onset refractory status epilepticus (NORSE) is a neurological emergency that not only occurs in patients with no previous history of neurological disease but also often has unknown etiology (Hirsch et al., 2018). The prognosis is extremely poor, as 62% of the 130 patients had poor outcomes with a 22% mortality rate (Gaspard et al., 2015). The pathophysiological mechanisms underlying NORSE are currently unknown and there are no standard international guidelines for treatment. Thus, a current research focus is the identification of variables that affect outcomes in NORSE patients.

Antiviral treatment was one of the predictive factors possibly due to the effectiveness of specific therapies for validated etiologies. Although autoimmune disorders are the most common etiological diagnoses (37%) for NORSE, little is known on other etiologies apart from infectious agents (8%) which are mainly viral (Yu et al., 2021). The diagnosis of NORSE does not exclude any particular virus to prevent varying disease definitions due to the capacities for viral identification (Gaspard et al., 2015). Moreover, the causal role of testing for certain viruses is unclear. NORSE patients often have an unclear etiology upon admission, which leads to a differential diagnosis of autoimmune encephalitis or viral encephalitis. As a results, attempted antiviral therapy, primarily acyclovir, is provided to most patients. Our study diagnosed patients with viral encephalitis mainly based on clinical manifestations and ancillary tests with negative cerebrospinal fluid polymerase chain reaction (PCR). Literatures suggest that initially PCR-negative status epilepsy can become PCR-positive after a period of illness, so antiviral therapy may still be effective (Buerger et al., 2015; Roberts et al., 2021). Case reports also propose that NORSE may be a precursor manifestation of herpes simplex viral encephalitis, even in PCR-negative cases and seizures may only cease after treating the etiology (Verma et al., 2013). However, lumbar puncture is often not repeated to confirm the etiologic agent due to the disease’s rapid progression. Our study found an independent effect of antiviral therapy on outcome after first univariate and then multifactorial analyses, ruling out the effect of other interfering or confounding factors. As the causal role of viral positivity is currently unknown, it is necessary to design prospective studies with periodic repeat lumbar puncture data to determine the role of antiviral therapy.

Current treatment of NORSE can be divided into two axis: disease-modifying treatments through immunotherapy and administration of appropriate antiepileptic drugs (AEDs) to terminate seizures (Jang et al., 2020). Current management of status epilepsy varies with region, and case studies are often on the selection of the best AEDs. Yet AEDs treatment is often disappointing with many patients requiring multiple anesthetics to regain control—status epilepticus often reoccurs upon discontinuation of the drug (Kramer et al., 2011; Gaspard et al., 2015; van Baalen et al., 2017). In this study, almost all patients were on one or more anesthetic drugs. The use of anesthetic agents is often associated with adverse outcomes, whose risk increases significantly with the number of anesthetic agents used, either sequentially or concurrently (Gaspard et al., 2015) and this corroborated our findings. Relatedly, adult SE patients under intravenous anesthetic therapies had high rates of infections and an increased risk of death (Hocker et al., 2013; Sutter et al., 2014; Marchi et al., 2015; Hocker, 2019), casting doubt on the efficacy of anesthetics for the treatment of persistent epilepsy. The severity and duration of SE is hypothesized as the main proponent for the use of anesthetic drugs and resultant high complications and mortality (Towne et al., 1994). Patients with refractory status epilepsy (RSE) who were under high doses of anesthetics had better outcomes than those under low doses further supporting the hypothesis that the severity of RSE itself, and not the use of anesthetics (Fernandez et al., 2014), is the main determinant of adverse outcomes. In the initial phase of our study, we attempted to conduct a subgroup analysis based on the types of anesthetics used. However, due to the limited size of our sample, this endeavor did not yield effective statistical analysis. Therefore, future research will require a larger sample size to thoroughly investigate the impact of different anesthetics on the prognosis of patients with NORSE.

Mechanical ventilation was a risk factor for adverse outcomes in NORSE patients, corroborating mechanical ventilation during hospitalization as an independent risk factor for adverse outcomes in RSE and the development of post-autoimmune encephalitis epilepsy (PAEE) (Lowenstein, 1999; Li et al., 2009; Chen et al., 2021). Over 90% of RSE cases required mechanical ventilation, a third of which eventually required tracheotomies. The longer the duration of mechanical ventilation, the higher the resultant mortality rate (Hocker et al., 2013). In a coma, tracheal intubation is used to protect the airwaves of patients with refractory persistent epilepsy (Zeidan et al., 2021). Yet patients who present this degree of impaired consciousness often have poor outcomes.

The peripheral blood neutrophil-to-lymphocyte ratio (NLR) is an increasingly recognized biomarker of systemic inflammation for a variety of disease states (Celikbilek et al., 2013; Demirci et al., 2016; Mercan et al., 2016; Qin et al., 2016). In NORSE autoimmune pathogenesis is more common than viral infections (Jang et al., 2020). Therefore, NORSE is a potential autoimmune epilepsy requiring aggressive immunotherapy following assessment of the infection (Jang et al., 2020). Based on relationships between autoimmune encephalitis and NLR, the best threshold for predicting severe disability (mRS > 2) was NLR > 4.82 (AUC 0.875) (Zeng et al., 2019). Here, using univariate and multivariate analyses, we also identified NLR > 4.82 as an independent risk factor. For autoimmune encephalitis patients, each 1-unit increase in NLR was associated with a 1.3-fold increase in the odds of treatment failure (Broadley et al., 2020). The similarity in clinical features and laboratory results between NORSE patients with an autoimmune etiology and those without a known etiology suggests that we can identify more autoimmune etiologies, especially with the discovery of autoantibodies (Gaspard et al., 2015).

To accurately predict the risk of adverse outcomes in NORSE patients, we constructed a nomogram based on the aforementioned factors and internally validated it. The calibration curve was close to the ideal curve and there was no statistically significant difference between the predicted and actual values. From ROC curve analysis, the area under the curve for the model, which predicted the occurrence of adverse outcomes in NORSE patients was 0.912 (95% CI: 0.844–0.980), indicating that the model had good consistency and discrimination in predicting risk.

This study, utilizing a nomogram predictive model based on clinical data from NORSE patients, effectively aids clinicians in assessing the prognosis of NORSE patients and provides reference for further interventions. However, there are several limitations to our research. Firstly, our study was based on clinical data from a single center to develop the nomogram model and only underwent internal validation, lacking verification against external clinical data. Therefore, we need to collect a broader dataset to further validate its applicability in different clinical settings. Secondly, the limited sample size of clinical data in our study resulted in high variability, increasing the uncertainty in risk prediction for individual patients and diminishing the ability to generalize the study results to a broader population. This limitation also restricted the depth and breadth of subgroup analyses, such as those involving different types of anesthetic drugs. Expanding the sample size for more extensive validation would be beneficial. If future research could increase the sample size and design prospective studies or subgroup analyses to further validate and confirm the model’s effectiveness and reliability, it would provide better clinical application and promotional value. Additionally, considering the potential fluctuations in the condition of NORSE patients, incorporating follow-up data and further survival analysis, if feasible, could be more meaningful for accurately assessing patient outcomes.

## 5 Conclusion

In conclusion, independent risk factors for adverse outcomes in NORSE are antiviral therapy, the number of rounds of IVIg, the number of anesthetics, mechanical ventilation and high NLR score. The nomogram for predicting the risk of poor outcomes had good consistency and discrimination in predicting risk and can thus assist clinical care providers to assess outcomes for NORSE patients. However, this single-center study had a small sample size and the model was only validated internally. Therefore, the discrimination and accuracy of this model should be further confirmed by analyzing data from multiple centers, increasing the sample size and adopting a combination of internal and external validations.

## Data availability statement

The raw data supporting the conclusions of this article will be made available by the authors, without undue reservation.

## Ethics statement

The studies involving humans were approved by the Ethics Committee of the First Affiliated Hospital of Sun Yat-sen University. The studies were conducted in accordance with the local legislation and institutional requirements. Written informed consent for participation was not required from the participants or the participants’ legal guardians/next of kin in accordance with the national legislation and institutional requirements.

## Author contributions

QL: Data curation, Formal analysis, Investigation, Methodology, Project administration, Resources, Software, Writing – original draft. RL: Funding acquisition, Investigation, Methodology, Writing – original draft. MS: Methodology, Project administration, Resources, Writing – original draft. ZW: Project administration, Resources, Software, Writing – original draft. HF: Funding acquisition, Supervision, Validation, Visualization, Writing – review & editing. HZ: Conceptualization, Data curation, Formal analysis, Supervision, Validation, Visualization, Writing – review & editing.
